# In-Depth Characterisation of Real-World Advanced Melanoma Patients Receiving Immunotherapies and/or Targeted Therapies: A Case Series

**DOI:** 10.3390/cancers14112801

**Published:** 2022-06-04

**Authors:** Saira Sanjida, Brigid Betz-Stablein, Victoria Atkinson, Monika Janda, Ramez Barsoum, Harrison Aljian Edwards, Frank Chiu, My Co Tran, H Peter Soyer, Helmut Schaider

**Affiliations:** 1 Centre for Health Services Research, Faculty of Medicine, The University of Queensland, Woolloongabba, QLD 4102, Australia; s.sanjida@uq.edu.au (S.S.); m.janda@uq.edu.au (M.J.); 2 The University of Queensland Diamantina Institute, Dermatology Research Centre, The University of Queensland, Woolloongabba, QLD 4102, Australia; r.barsoum@uq.edu.au (R.B.); harrison.edwards@uq.net.au (H.A.E.); f.chiu@uq.edu.au (F.C.); myco.tran@uq.edu.au (M.C.T.); p.soyer@uq.edu.au (H.P.S.); h.schaider@uq.edu.au (H.S.); 3 Cancer Care Services, Princess Alexandra Hospital, Woolloongabba, QLD 4102, Australia; victoria.atkinson@health.qld.gov.au; 4 Dermatology Department, Princess Alexandra Hospital, Woolloongabba, QLD 4102, Australia

**Keywords:** advanced melanoma, immunotherapy, targeted therapy, survival, real-world setting

## Abstract

**Simple Summary:**

Immunotherapies and targeted therapies have led to improved melanoma survival in clinical trial settings. While this is also true in real-world settings, it is much less studied. Clinical trials have strict inclusion criteria and, therefore, a relatively homogenous set of participants. However, this is not the case in real-world settings, and the differences in characteristics are rarely described in association with survival. In this 3D total-body photography imaging study, we describe the characteristics and clinical outcomes of 41 study participants who received immuno- and/or targeted therapies for metastatic melanoma in a real-world setting. After a median of 39 months follow-up, 59% (*n* = 24/41) of the participants were alive. Our sample size was too small to detect significant differences between patient characteristics; however, despite the majority of our participants having multiple comorbidities, survival was similar to previous reported clinical trials and other real-world settings.

**Abstract:**

Immunotherapies and targeted therapies have shown significant benefits for melanoma survival in the clinical trial setting. Much less is known about the characteristics and associated outcomes of those receiving such therapies in real-world settings. This study describes the characteristics of patients with advanced melanoma receiving immuno- and/or targeted therapies in a real-world setting. This prospective cohort study enrolled participants aged >18 years, diagnosed with advanced melanoma and currently undergoing immuno- and/or targeted therapies outside a clinical trial for follow-up with three-dimensional (3D) total-body imaging. Participants (*n* = 41) had a mean age of 62 years (range 29–86), 26 (63%) were male and the majority (*n* = 26, 63%) had ≥2 comorbidities. After a median of 39 months (range 1–52) follow-up, 59% (*n* = 24/41) of participants were alive. Despite multiple co-morbidities, the survival of participants with advanced melanoma treated using immuno- and/or targeted therapies was similar or better in our real-world setting compared to those treated in clinical trials using similar therapies. Larger studies powered to evaluate phenotypic and socio-economic characteristics, as well as specific comorbidities associated with survival in a real-world setting, are required to help determine those who will most benefit from immuno- and/or targeted therapies.

## 1. Introduction

Australia and New Zealand have the highest incidence rates of melanoma in the world [1,2]. The risk of developing melanoma is associated with multiple characteristics, including socio-demographic (e.g., age, country), clinical (e.g., melanoma history), environmental (e.g., sun exposure), behavioural (e.g., use of sun protection) and phenotypical (e.g., hair, skin colour) [3,4,5]. When diagnosed early, the 5-year survival rate is typically greater than 90% [6]. However, until recently, there was a reduced survival rate of 18% for those diagnosed with advanced metastatic melanoma [3].

Over the past decade (2010–2020), there have been significant improvements in the treatment options for advanced melanoma, particularly through the use of immunotherapy (e.g., programmed cell death 1, PD-1; cytotoxic T-lymphocyte-associated protein 4, CLTA-4) and targeted therapy (e.g., mitogen-activated protein kinase, MEK; B-Raf proto-oncogene serine/threonine kinase, BRAF), resulting in improved survival [7]. These therapies are currently considered as the standard of care and the first-line treatment for advanced stage melanoma, given the rapid treatment response, efficacy against brain metastases and long-term treatment benefits [8]. A recent review [9] reported that 5 year survival of metastatic melanoma patients in clinical trials was 1 in 2 for those receiving a combination of immunotherapy [10] and more than 1 in 3 when receiving a combination of BRAF/MAPK kinase targeted therapy [11] or single-agent PD-1 blockade [12]. This is a stark improvement to the <5% survival of metastatic melanoma patients 10 years ago [13]. However, clinical trials follow strict inclusion and exclusion criteria where age, health and accessibility criteria may not reflect the true heterogeneity of people treated for metastatic melanoma in the real-world.

Several studies have been conducted in real-world settings to determine the survival outcomes after immuno- and/or targeted therapies. A Dutch study evaluated 1004 advanced melanoma patients treated between 2014–2017 who were ineligible for clinical trials and found poorer overall survival (8.8 vs. 23 months). The study found poorer survival was associated with Eastern Cooperative Oncology Group Performance Score ≥ 2, brain metastases and lactate dehydrogenase > 500 U/L [14]. However, smaller studies have reported survival rates more similar to those of clinical trials, including a US study (*n* = 484) with an overall survival rate of 20.7 months [15] and a Slovenian study (*n* = 116) with overall survival of 33 months in advanced cutaneous melanoma patients [16]. A German real-world study showed, for advanced melanoma patients with mutant BRAF (V600E/K), a higher overall survival of 29 months for those receiving PD-1 compared with 12 months for those receiving dual MAPKi [17]. Many of these studies required participants to be receiving a specific immunotherapy, whereas there is sparse published research reflecting the real-world scenario where participants often receive multiple treatments (immuno- and/or targeted therapies), and in combinations that change over time. This prospective cohort study was designed to use 3D total-body photography to observe changes in naevi over time and provided the opportunity to comprehensively describe the characteristics, treatment and survival of a series of advanced-stage melanoma patients receiving immuno- and/or targeted therapies in a real-world setting in Queensland, Australia.

## 2. Methods and Materials

### 2.1. Study Setting and Participant Recruitment

This prospective cohort study enrolled participants with advanced melanoma at the Dermatology Outpatients Department at the Princess Alexandra Hospital, Queensland, Australia, between June 2016 and December 2017. Participants were eligible for inclusion if they were >18 years of age, diagnosed with stage III–IV melanoma and were then undergoing treatment with immuno- and/or targeted therapy. Concurrent enrolment in a clinical trial during 12 months’ follow-up excluded participants from participating in this observational study. Participants were actively followed for 12 months with 3D total-body imaging to study the natural history of naevi in participants undergoing immuno- and/or targeted therapy. Inactive observation ended on 31 October 2020 where further treatment and survival outcome data were collected from electronic medical records.

### 2.2. Data Collection

Socio-demographic, environmental, phenotypic and clinical characteristics were collected by clinical research assistants using standard questionnaires. The socio-demographic characteristics collected including gender, age, private health insurance and highest educational attainment. Participants were asked about their sun exposure patterns, such as whether their occupations after leaving school and their sport and leisure activities were indoors or outdoors. The participants’ natural hair colour at age 21 and severity of freckling were recorded. Freckling density was assessed on three body sites, the face, the dorsum of the right arm and the shoulders, and rated for level of freckling by clinic staff from 1 for ‘none’ to 4 for ‘severe’. The composite of these was given an overall freckling score [18] and re-categorised into none/light (1–3), mild (4–6), medium (7–9) and severe (10–12). Body mass index (BMI) was calculated and categorised according to World Health Organization (WHO) [19]. Three-dimensional total-body images along with dermoscopic images of all naevi > 5 mm were collected every 4 months. For those with longitudinal follow-up, change was evaluated across all available sequential dermoscopic (range 2–4) images by a dermatology registrar and confirmed by a senior dermatologist. Change was defined as a change > 15% in size, shape, colour, profile or naevus dermoscopic pattern and categorised as decreasing, stable or increasing. Clinical staff collected melanoma history and comorbidity information from electronic medical records and included details of melanoma diagnosis (such as primary melanoma location, multiple primary melanomas diagnosed), time since diagnosis (the first and the most recent melanoma if applicable) and details of treatment received during study timeline.

### 2.3. Statistical Analyses

Descriptive statistics were used to determine the frequency and distribution of participants’ socio-demographic, clinical, environmental, behavioural and phenotypic characteristics. Continuous variables were summarised as means with standard deviation (SD) or medians (range) as appropriate, with categorical variables as frequency and proportion. Survival status was calculated as of the censored date (OS) of 31 October 2020. Follow-up time was calculated from the time between baseline visit to censored date according to the survival status of participants. Median overall survival time could not be calculated as > 50% of participants were still alive at the end of the observation period. All statistical analyses were performed in SPSS 27.0 (Statistical Package for the Social Sciences software, IBM SPSS Statistics for Windows, Chicago, IL, USA) or R [20].

## 3. Results

Forty-three participants were enrolled in the study between June 2016 and December 2017, and 2 were excluded as no record was found of the participants receiving immuno- and/or targeted therapies. Therefore, 41 participants were followed for a median of 39 months (range 1–52 months).

### 3.1. Baseline Characteristics

The participants had a median age of 65 years (range 29–86), and the majority were male (*n* = 26, 63%) (Table 1). The minority had private health insurance (*n* = 10, 29%), and just over half of participants had a post-high school qualification (*n* = 23, 56%). Approximately half of participants (*n* = 23, 56%) worked mainly indoors, with most participants spending their leisure time outdoors (*n* = 28, 68%). About half reported dark brown or black hair at age 21 (*n* = 21, 51%), with innate skin colour classified as fair in the majority (*n* = 31, 76%). Approximately half of the participants had no or only mild freckling on their arms, shoulders and faces (*n* = 18, 44%), while the remainder had medium to severe freckling (*n* = 23, 56%). Median naevus count > 5 mm was 8 (range 1–60.) Based on BMI, one participant was underweight, 15 (37%) were considered normal weight, 10 (24%) overweight and 15 (37%) obese.

### 3.2. Comorbidities

The majority of participants had multiple comorbidities (*n* = 26, 63%) (Table 1). The most common comorbidity was hypertension (*n*= 14, 34%), followed by hypercholesterolemia or hyperlipidemia (*n* = 10, 22%). A considerable number of participants also reported cardiovascular disease (*n* = 8, 19%), diabetes mellitus (*n* = 8, 19%), cancer other than skin cancer (*n* = 4, 8%) and liver disease (*n* = 4, 9%).

### 3.3. Melanoma History

About half of participants (*n* = 22, 54%) had only one previous melanoma, while 17 participants (*n* = 41%) had multiple prior melanomas (range 2–7) (Table 1). The median time since diagnosis of the first and most recent primary melanoma was 6 years (2–24) and 5 years (1–24), respectively. The most recent primaries were located primarily on the chest/abdomen (*n* = 12, 29%), followed by the head and neck (*n* = 10, 24%), lower limbs (*n* = 9, 22%), back (*n* = 4, 10%) and upper limbs (*n* = 3, 7%). Three participants (7%) had unknown primaries. Most participants had stage IV metastatic melanoma (*n* = 36, 88%), while the remaining 5 (12%) had stage III melanoma with only lymph metastases reported. Twenty-two per cent (*n* = 9) reported brain metastases.

### 3.4. Treatments Received for Advanced Melanoma

Around two-thirds of participants (*n* = 24, 59%) received two or more therapies (Table 2). PD-1 blocker was the first line of treatment for the majority of patients (63%, *n* = 26), followed by the combination of BRAF and MEK inhibitors (*n* = 9, 22%). The most common immuno- and/or targeted therapies prescribed for the treatment of advanced melanoma in this case series were PD-1 blocker (pembrolizumab, *n* = 29), BRAF inhibitor (dabrafenib, *n* = 14) and MEK inhibitor (trametinib *n* = 14). Treatment regimens were commonly adjusted due to progressive disease (*n* = 19), toxicity or other adverse effects (*n* = 8). At the censoring date (October 2020), five participants were still receiving treatment (Table 2).

### 3.5. Overall Survival

After a median follow-up time of 39 months, 24 (58%) participants were alive. The sample size did not allow testing for significant differences between those alive and those deceased; however, here we summarise characteristics showing a difference in survival at proportions greater than 10% (Table 1). A slightly higher proportion of females survived compared with males (67% vs. 54%). A higher proportion of those with private health insurance (80%) survived, compared with those without private health insurance (54%) and those that did not report whether they had insurance (43%). Those who worked mainly indoors as opposed to mainly outdoors or both indoors and outdoors showed higher rate of survival (67% vs. 52%). Similarly, a higher proportion of those who spent their leisure time ‘mainly indoors’ or ‘both indoors and outdoors’ survived opposed to those who spent their leisure time ‘mainly outdoors’ (69% vs. 54%). Participants with red/auburn/blonde/light brown hair showed higher levels of survival compared to those with dark brown or black hair (76% vs. 40%). A higher proportion of those with fair innate and fair facultative skin colour survived than those with medium to olive skin tones (respectively, 61% vs. 50%, 69% vs. 52%). With regards to BMI, a higher proportion classified as obese (67%) survived compared to those who were considered normal (63%) or overweight (40%). While the number of co-morbidities showed similar levels of survival, those with hypertension (*n* = 16) showed a higher level of survival compared to those without (50% vs. 37%), and those with diabetes showed a lower rate of survival (38% vs. 64%). Similar rates of survival were seen in those with stage III and stage IV melanoma (60% vs. 62%); however, it should be noted that only five (12%) participants included in the study were stage III. A higher proportion of participants without brain metastasis survived compared to participants with brain metastasis (69% vs. 33%).

### 3.6. Naevus Change

Of the 41 participants enrolled in this study, 28 (68%) completed a minimum of two imaging visits, with a median follow-up time of 11 months (range 3–19). Demographics of this subset are presented in Supplementary Table S1. Overall, 387 naevi > 5 mm were imaged, with each participant having a median of 7 naevi (range 1–59) (Table 3). The majority of naevi (*n* = 259, 67%) did not change, with 114 (29%) decreasing and 15 (4%) increasing. Per person, this corresponded to a median of 6 (range 0–51) stable and 1 (range 0–26) changing naevi, with 0 increasing (range 0–5) and 1 (range 0–26) decreasing. Within each individual, the mean proportion of changing naevi > 5 mm was 34% (SD 36). Given the small sample and innate bias, those who completed the longitudinal follow-up were healthier and/or responded better to treatment, we did not compare change by survival status; however, numbers are provided in Table 3 for future meta-analyses.

## 4. Discussion

This prospective cohort study originally designed to study the natural history of naevi in individuals receiving immune- and/or targeted therapies for metastatic melanoma allowed for the comprehensive description of characteristics, treatment and survival in an under-studied population in a real-world setting. At the end of the median follow-up of 39 months, 58% (*n* = 24/41) of participants remained alive. This is similar or higher than the proportions reported in clinical trial settings [10].

Treatment response and survival in patients with metastatic melanoma is likely a complex interaction between phenotypic, genetic and sociodemographic characteristics. While this study was not originally designed, and therefore powered, to test for significance between survival and such variables, it may point towards characteristics that should be investigated in larger studies. The majority of variables, including age, showed similar proportions of patients both alive and deceased (within ± 10%). Contradicting results have been seen with respect to age, with some showing increased survival in those under 60 [14] and 64 [21], respectively, while others show no difference in overall survival [15,22,23,24]. In our participants, a slightly higher proportion of females remained alive at the end of our study. While in men, lower awareness and self-detection of melanoma, less frequent skin monitoring and higher sun exposure are all possible reasons for both late diagnosis of melanoma and reduced survival in males [25], when tested, no significant difference in survival was seen between sexes in other real-world studies [14,15,22,26,27]. Few studies have investigated phenotypic, sun behaviour-related characteristics or socio-economic status. A French study (*n* = 87) has explored survival outcomes for real-world data with participant characteristics since the new melanoma immuno- and/or targeted therapies have become available and showed no significant association between number of naevi, phototype and sun exposure characteristics [23]. However, we saw slightly higher proportions of survival in those with lower occupational and leisure sun exposure and those with lighter facultative and innate skin tone. This difference could easily be explained by the different measures of sun exposure and phototype or merely be an artefact due to the small sample size. Our study also showed a higher proportion of those with private health insurance survived compared to those who did not report having private health insurance, but as this is contrary to an earlier Australian study of PBS-subsidised ipilimumab [22], it may merely reflect our sample size.

In contrast to the clinical trial setting, the majority of our participants had more than one comorbidity. A recent retrospective Dutch study of 2216 metastatic melanoma patients ≥ 65 years under immuno- and/or targeted therapies showed no association with age, sex or number of comorbidities with respect to response or survival after a median follow-up of 0.7 years [26]. A smaller Italian study (*n* = 174) of metastatic melanoma patients ≥ 75 years receiving Anti-PD1 antibodies also showed no association with the number of comorbidities relating to either progression-free or overall survival [27]. While several studies have considered the effects of the number of comorbidities, few have looked at the effects of specific comorbidities. While we cannot draw any conclusions given the limited sample size of our study, our study indicates that specific comorbidities may have opposing effects, with potential associations with both improved and decreased survival. Interestingly, a recent review [28] summarised several studies where obesity was shown to be associated with improved overall survival of metastatic melanoma patients on immunotherapies. Although we did not have the power to test for a significant association, we did see a higher proportion of those classified as obese survive. In two studies, this association was stronger [29] or seen only in males [30]. Those with brain metastasis had a lower proportion survive in our study, which is consistent with other real-world studies of those receiving immuno- and/or targeted therapies [14,15,24,26].

With respect to naevus change in those undergoing immuno- and/or targeted therapies, several case reports have described hypopigmentation of naevi, regression/involution of naevi with and without the halo effect and, in a few cases, appearance of new naevi [31,32,33,34,35,36,37,38]. An Italian study of 11 patients receiving dabrafenib observed the appearance of new naevi in 4/11 patients, and hyperkeratosis/hyperpigmentation in 6/11 patients [39]. An Australian study compared naevus changes in patients (*n* = 40) receiving four different combinations of immuno- and targeted therapies to controls (*n* = 10), and observed naevus darkening most often under BRAF inhibitor therapy, with naevus hypopigmentation more common in those under a combination of dabrafenib and trametinib [40]. There was also lesion lightening observed in those receiving anti-PD1 therapies with and without ipilimumab. In some cases, regression of naevi has been observed alongside regression of both primary [31] and metastatic melanomas [33,36,37], and it has been suggested that regression of many naevi may be a prognostic marker, highlighting the importance of including dermatologist follow-up in clinical trials and the real-world setting [33,35,40]. Our study supports the above findings, largely showing regressing naevi but also a small number of increasing naevi.

While our sample size was small (*n* = 41), our participants were followed prospectively and our study was not limited to a specific treatment. The small sample size did limit statistical power and, therefore, we were unable to test for statistical significance. A limitation of this study is that health-conscious participants were more likely to participate in this study, allowing the potential for health awareness bias [41]. This may explain the longer survival times observed in this study or, more likely, reflects the innate self-selection bias in that patients with a short life expectancy are less likely to be referred to a dermatology department for participation in research studies. In addition, as this study was not designed to evaluate survival, other important characteristics such as LDH and frailty score were not collected. Nonetheless, this prospective case series shows that, despite a higher rate of co-morbidities, participants in a real-world setting can have similar survival times compared to those in the clinical trial setting. However, given our sample size, even descriptive statistics can be misleading. Therefore, we suggest this study not be used to draw conclusions but rather to inform future data collection in real-world clinical studies. Specifically, collecting data such as demographic and phenotypic (including naevus change) characteristics, as well as specific comorbidities, in larger survival studies powered for both univariate and multivariable analysis may help determine those who will most benefit from immune- and/or targeted therapy treatment.

## Figures and Tables

**Table 1:** Baseline sociodemographic and clinical characteristics (frequency, column percentages) of participants with advanced melanoma by survival status (frequency, row percentages).

	*n* = 41 (%)	Alive *n* = 24 (59%)	Deceased *n* = 17 (41%)
**Socio-demographic characteristics, *n* (%)**			
**Age ^1^: Mean (SD)**	62 [14]	61 [16]	63 [13]
**Category**			
≤65 years >65 years	21 (51) 20 (49)	12 (57) 12 (60)	9 (43) 8 (40)
**Gender**			
Male Female	26 (63) 15 (37)	14 (54) 10 (67)	12 (46) 5 (33)
**Private health insurance**			
Yes	10 (24)	8 (80)	2 (20)
No	24 (59)	13 (54)	11 (46)
Not Reported	7 (17)	3 (43)	4 (57)
**Education**			
Higher school or less	18 (44)	9 (50)	9 (50)
Post-high school qualification	23 (56)	15 (65)	8 (35)
**Environmental exposure, *n* (%)**			
**Occupations since leaving school**			
Mainly indoors	18 (44)	12 (67)	6 (33)
Mainly outdoors/both indoors and outdoors	23 (56)	12 (52)	11 (48)
**Overall sports and leisure activity**			
Mainly indoors/both indoors and outdoors	13 (32)	9 (69)	4 (31)
Mainly outdoors	28 (68)	15 (54)	13 (46)
**Phenotype characteristics, *n* (%)**			
**Naevi Count (>5 mm)**			
Median (range)	8 (1–60)	8 (1–59)	9 (3–60)
**Natural hair colour at age 21**			
Red/auburn/blonde/light brown	21 (51)	16 (76)	5 (24)
Dark brown/black	20 (49)	8 (40)	12 (60)
**Innate skin colour**			
Fair	31 (76)	19 (61)	12 (39)
Medium or olive	10 (24)	5 (50)	5 (50)
**Facultative skin colour**			
Fair	16 (39)	11 (69)	5 (31)
Medium or olive	25 (61)	13 (52)	12 (48)
**Freckling score**			
Nil/mild (0–6)	18 (44)	11 (61)	7 (39)
Mild/severe (7–12)	23 (56)	13 (57)	10 (43)
**Body mass index (kg/m^2^)**			
Normal (≤24.9) ^2^	16 (39)	10 (63)	6 (37)
Overweight (25.0–29.9)	10 (24)	4 (40)	6 (60)
Obese (≥30.0)	15 (37)	10 (67)	5 (33)
**Clinical characteristics**			
**Number of comorbidities**			
None	5 (12)	3 (60)	2 (40)
1	10 (25)	5 (50)	5 (50)
2 or more	26 (63)	16 (62)	10 (38)
**Comorbidities ^3^**			
Hypertension			
Yes	14 (34)	7 (50)	7 (50)
No	27 (66)	17 (37)	10 (63)
Hypercholesterolemia or hyperlipidemia			
Yes	10 (22)	6 (60)	4 (40)
No	31 (78)	18 (58)	13 (42)
Cardiovascular disease			
Yes	8 (20)	4 (50)	4 (50)
No	33 (80)	20 (61)	13 (39)
Diabetes mellitus			
Yes	8 (20)	3 (38)	5 (62)
No	33 (80)	21 (64)	12 (36)
**Melanoma history**			
**Number of primary melanomas**			
1	22 (54)	14 (64)	8 (36)
2–7	17 (41)	10 (59)	7 (41)
**Time since diagnosis of most recent primary melanoma (*n*= 36) ^4^**			
≤5 years	21 (58)	12 (57)	9 (43)
5 to ≤10 years	11 (30)	8 (73)	3 (27)
≥11 years	4 (12)	3 (75)	1 (8)
**Time since diagnosis metastatic melanoma**			
≤1 year	16 (39)	0	16 (100)
2 to 3 years	13 (32)	12 (92)	1 (8)
≥4 years	12 (29)	12 (100)	0
**Melanoma stage**			
Stage III	5 (12)	3 (60)	2 (40)
Stage IV	36 (88)	21 (58)	15 (42)
**Brain metastasis**			
Yes	9 (22)	3 (33)	6 (67)
No	32 (78)	21 (66)	11 (34)

NR: Not reported, SD: standard deviation. ^1^ Median: 65 years: range: 29 to 86 years; ^2^ includes 1 underweight (BMI = 16.8); ^3^ percentages do not add up to 100 as participants could have multiple co-morbidities; ^4^ Median: 5 years: range: 1 to 24 years

**Table 2:** Immunotherapy and/or targeted therapy delivered to the participants with advanced melanoma at any time throughout follow-up. Percentages do not add up to 100 as participants could receive multiple treatments over the follow-up period.

Type of Treatment Delivered	*n* = 41 (%)	Alive, *n* = 24 (%)	Deceased, *n* = 17 (%)	
**Immunotherapy at any time**				
PD-1 blocker				
Pembrolizumab	29 (71)	17 (71)	12 (71)	
Nivolumab	13 (32)	6 (25)	7 (41)	
Atezolizumab	1 (2)	0	1 (6)	
CTLA-4 blocker				
Ipilimumab	13 (32)	7 (29)	6 (35)	
**Targeted therapy at any time**				
MEK inhibitor				
Trametinib	14 (34)	8 (33)	6 (35)	
BRAF inhibitor				
Dabrafenib	14 (34)	8 (33)	6 (35)	
Vemurafenib	3 (7)	-	3 (18)	
MEK inhibitor				
Cobimetinib	2 (5)	-	2 (11)	
**Total number of melanoma treatments received during follow-up**				
One	17 (41)	1 (4)	6 (35)	
Two or more	24 (59)	13 (54)	11 (65)	
**Combination of immune- and targeted therapy at one time**	3 (7)	2 (8)	1 (6)	
**Continuation of treatments until October 2020**	5 (12)	5 (21)	-	
Line of treatment * **(June 2016–October 2020)**	**PD-1 blocker**	**PD-1 and CTLA-4 blockers combination**	**BRAF and MEK inhibitors combination**	**Other** **^¶^**
First-line treatment (*n* = 41)	26 (63)	4 (10)	9 (22)	2 (5)
Second-line treatment (*n* = 19)	4 (21)	6 (31)	4 (21)	5 (26)
Third-line and following treatment (*n* = 3)	1 (33)	1 (34)	1 (33)	0

**^¶^** Included ipilimumab, dabrafenib and trametinib alone. * The line of treatment was determined from the delivery of immuno- and/or targeted therapies to the participants with advanced melanoma after enrolment in this study according to date. Percentages presented by row.

**Table 3:** Naevus change >5 mm for each patient.

	*n* = 28 (%)	Alive *n* = 21 (75%)	Deceased *n* = 7 (25%)
**Total Body Naevus Count (>5 mm)**			
Median (range)	7 (1–59)	7 (1–59)	7 (4–45)
**Number of Stable Naevi**			
Median (range)	6 (0–51)	4 (0–51)	7 (0–19)
**Number Naevi Changing**			
Median (range)	1 (0–26)	1 (0–24)	3 (0–26)
**Number of Increasing Naevi**			
Median (range)	0 (0–5)	0 (0–5)	0 (0–4)
**Number of Decreasing Naevi**			
Median (range)	1 (0–26)	1 (0–19)	1 (0–26)
**Proportion of Stable Naevi**			
Mean (SD)	66 (37)	65 (37)	66 (39)
**Proportion of Changing Naevi**			
Mean (SD)	34 (37)	35 (37)	34 (39)
**Proportion of Increasing Naevi**			
Mean (SD)	4 (15)	2 (4)	12 (30)
**Proportion of Decreasing Naevi**			
Mean (SD)	30 (34)	33 (37)	22 (25)

## Data Availability

Due to privacy and ethical concerns, the data that support the findings of this study are available on request from the corresponding author.

## Supplementary Materials

The following supporting information can be downloaded at: https://www.mdpi.com/article/10.3390/cancers14112801/s1, Table S1: Baseline sociodemographic and clinical characteristics (Fre-quency, column percentages) of participants with and without longitudinal follow up (Frequency, row percentages).

## References

[1] Arnold M., de Vries E., Whiteman D.C., Jemal A., Bray F., Parkin D.M., Soerjomataram I. (2018). Global burden of cutaneous melanoma attributable to ultraviolet radiation in 2012. Int. J. Cancer.

[2] Whiteman D.C., Green A.C., Olsen C. (2016). The Growing Burden of Invasive Melanoma: Projections of Incidence Rates and Numbers of New Cases in Six Susceptible Populations through 2031. J. Investig. Dermatol..

[3] Johnson M.M., Leachman S.A., Aspinwall L.G., Cranmer L.D., Curiel-Lewandrowski C., Sondak V.K., Stemwedel C.E., Swetter S.M., Vetto J., Bowles T. (2017). Skin cancer screening: Recommendations for data-driven screening guidelines and a review of the US Preventive Services Task Force controversy. Melanoma Manag..

[4] Olsen C.M., Pandeya N., Thompson B.S., Dusingize J.C., Green A.C., Neale R.E., Whiteman D.C., for the QSkin Study (2019). Association between Phenotypic Characteristics and Melanoma in a Large Prospective Cohort Study. J. Investig. Dermatol..

[5] Rastrelli M., Tropea S., Rossi C.R., Alaibac M. (2014). Melanoma: Epidemiology, risk factors, pathogenesis, diagnosis and classification. In Vivo.

[6] National Cancer Institute Cancer Stat Facts: Melanoma of the Skin 2019. https://seer.cancer.gov/statfacts/html/melan.html.

[7] Dummer R., Schadendorf D., Ascierto P.A., Larkin J., Lebbe C., Hauschild A. (2015). Integrating first-line treatment options into clinical practice: What’s new in advanced melanoma?. Melanoma Res..

[8] Luke J.J., Flaherty K.T., Ribas A., Long G.V. (2017). Targeted agents and immunotherapies: Optimizing outcomes in melanoma. Nat. Rev. Clin. Oncol..

[9] Jenkins R.W., Fisher D.E. (2020). Treatment of Advanced Melanoma in 2020 and Beyond. J. Investig. Dermatol..

[10] Larkin J., Chiarion-Sileni V., Gonzalez R., Grob J.-J., Rutkowski P., Lao C.D., Cowey C.L., Schadendorf D., Wagstaff J., Dummer R. (2019). Five-Year Survival with Combined Nivolumab and Ipilimumab in Advanced Melanoma. N. Engl. J. Med..

[11] Robert C., Grob J.J., Stroyakovskiy D., Karaszewska B., Hauschild A., Levchenko E., Chiarion Sileni V., Schachter J., Garbe C., Bondarenko I. (2019). Five-Year Outcomes with Dabrafenib plus Trametinib in Metastatic Melanoma. N. Engl. J. Med..

[12] Hamid O., Robert C., Daud A., Hodi F.S., Hwu W.J., Kefford R., Wolchok J.D., Hersey P., Joseph R., Weber J.S. (2019). Five-year survival outcomes for patients with advanced melanoma treated with pembrolizumab in KEYNOTE-001. Ann. Oncol..

[13] Dickson P.V., Gershenwald J.E. (2011). Staging and Prognosis of Cutaneous Melanoma. Surg. Oncol. Clin. N. Am..

[14] Van Zeijl M.C.T., Ismail R.K., de Wreede L.C., van den Eertwegh A.J.M., de Boer A., van Dartel M., Hilarius D.L., Aarts M.J., van den Berkmortel F.W., Boers-Sonderen M.J. (2020). Real-world outcomes of advanced melanoma patients not represented in phase III trials. Int. J. Cancer.

[15] Cowey C.L., Liu F.X., Boyd M., Aguilar K.M., Krepler C. (2019). Real-world treatment patterns and clinical outcomes among patients with advanced melanoma A retrospective, community oncology-based cohort study (A STROBE-compliant article). Medicine.

[16] Hribernik N., Boc M., Ocvirk J., Knez-Arbeiter J., Mesti T., Ignjatovic M., Rebersek M. (2020). Retrospective analysis of treatment-naive Slovenian patients with metastatic melanoma treated with pembrolizumab—real-world experience. Radiol. Oncol..

[17] Schilling B., Martens A., Foppen M.H.G., Gebhardt C., Hassel J.C., Rozeman E.A., Gesierich A., Gutzmer R., Kähler K.C., Livingstone E. (2019). First-line therapy-stratified survival in BRAF-mutant melanoma: A retrospective multicenter analysis. Cancer Immunol. Immunother..

[18] Duffy D., Box N., Chen W., Palmer J.S., Montgomery G., James M.R., Hayward N.K., Martin N., Sturm R.A. (2003). Interactive effects of MC1R and OCA2 on melanoma risk phenotypes. Hum. Mol. Genet..

[19] World Health Organization Body Mass Index 2019. http://www.euro.who.int/en/health-topics/disease-prevention/nutrition/a-healthy-lifestyle/body-mass-index-bmi.

[20] RC Team (2019). R: A Language and Environment for Statistical Computing.

[21] Moser J.C., Chen D., Hu-Lieskovan S., Grossmann K.F., Patel S., Colonna S.V., Ying J., Hyngstrom J.R. (2019). Real-world survival of patients with advanced BRAF V600 mutated melanoma treated with front-line BRAF/MEK inhibitors, anti-PD-1 antibodies, or nivolumab/ipilimumab. Cancer Med..

[22] Kim H., Comey S., Hausler K., Cook G. (2018). A real world example of coverage with evidence development in Australia—Ipilimumab for the treatment of metastatic melanoma. J. Pharm. Policy Pract..

[23] Bocquet-Tremoureux S., Scharbarg E., Nguyen J.M., Varey E., Quereux G., Saint-Jean M., Peuvrel L., Khammari A., Dreno B. (2019). Efficacy and safety of nivolumab in metastatic melanoma: Real-world practice. Eur. J. Dermatol. EJD.

[24] Howell A.V., Gebregziabher M., Thiers B.H., Paulos C.M., Wrangle J.M., Hunt K.J., Wallace K. (2020). Immune checkpoint inhibitors retain effectiveness in older patients with cutaneous metastatic melanoma. J. Geriatr. Oncol..

[25] Joosse A., de Vries E., Eckel R., Nijsten T., Eggermont A.M., Hölzel D., Coebergh J.W.W., Engel J. (2011). Gender Differences in Melanoma Survival: Female Patients Have a Decreased Risk of Metastasis. J. Investig. Dermatol..

[26] De Glas N., Bastiaannet E., Bos F.V.D., Mooijaart S., van der Veldt A., Suijkerbuijk K., Aarts M., Berkmortel F.V.D., Blank C., Boers-Sonderen M. (2021). Toxicity, Response and Survival in Older Patients with Metastatic Melanoma Treated with Checkpoint Inhibitors. Cancers.

[27] Ridolfi L., De Rosa F., Petracci E., Tanda E.T., Marra E., Pigozzo J., Marconcini R., Guida M., Cappellini G.C.A., Gallizzi G. (2020). Anti-PD1 antibodies in patients aged ≥75 years with metastatic melanoma: A retrospective multicentre study. J. Geriatr. Oncol..

[28] Smith L.K., Arabi S., Lelliott E.J., McArthur G.A., Sheppard K.E. (2020). Obesity and the impact on cutaneous melanoma: Friend or foe?. Cancers.

[29] McQuade J.L., Daniel C.R., Hess K.R., Mak C., Wang D.Y., Rai R.R., Park J.J., Haydu L.E., Spencer C., Wongchenko M. (2018). Association of body-mass index and outcomes in patients with metastatic melanoma treated with targeted therapy, immunotherapy, or chemotherapy: A retrospective, multicohort analysis. Lancet Oncol..

[30] Naik G.S., Waikar S.S., Johnson A.E.W., Buchbinder E.I., Haq R., Hodi F.S., Schoenfeld J.D., Ott P.A. (2019). Complex inter-relationship of body mass index, gender and serum creatinine on survival: Exploring the obesity paradox in melanoma patients treated with checkpoint inhibition. J. Immunother. Cancer.

[31] Grigore L.E., Ungureanu L., Bejinariu N., Seceac C., Vasilovici A., Senila S.C., Candrea E., Fechete O., Cosgarea R. (2019). Complete regression of primary melanoma associated with nevi involution under BRAF inhibitors: A case report and review of the literature. Oncol. Lett..

[32] McClenahan P., Lin L.L., Tan J.M., Flewell-Smith R., Schaider H., Jagirdar K., Atkinson V., Lambie D., Prow T.W., Sturm R.A. (2014). BRAFV600E mutation status of involuting and stable nevi in dabrafenib therapy with or without trametinib. JAMA Dermatol..

[33] Grassi S., Corsetti P., Moliterni E., Calvieri S. (2021). Regression of benign melanocytic nevi ipilimumab-induced in an adult patient affected by metastatic melanoma: Is it a sign of response to therapy?. Ital. J. Dermatol. Venerol..

[34] Wolner Z., Marghoob A., Pulitzer M., Postow M., Marchetti M. (2017). A case report of disappearing pigmented skin lesions associated with pembrolizumab treatment for metastatic melanoma. Br. J. Dermatol..

[35] Farinazzo E., Zelin E., Agozzino M., Papa G., Pizzichetta M.A., di Meo N., Zalaudek I. (2021). Regression of nevi, vitiligo-like depigmentation and halo phenomenon may indicate response to immunotherapy and targeted therapy in melanoma. Melanoma Res..

[36] Plaquevent M., Greliak A., Pinard C., Duval-Modeste A.-B., Joly P. (2019). Simultaneous long-lasting regression of multiple nevi and melanoma metastases after ipilimumab therapy. Melanoma Res..

[37] Libon F., Arrese J.E., Rorive A., Nikkels A.F. (2013). Ipilimumab induces simultaneous regression of melanocytic naevi and melanoma metastases. Clin. Exp. Dermatol..

[38] Schwager Z., Laird M.E., Latkowski J.-A. (2018). Regression of pigmented lesions in a patient with metastatic melanoma treated with immunotherapy. JAAD Case Rep..

[39] Dika E., Lambertini M., Fanti P.A., Piraccini B.M., Gurioli C., Ravaioli G.M., Chessa M.A., Gradassi A.T., Melotti B., Sperandi F. (2019). Sequential monitoring of pigmented lesions during dabrafenib treatment: A prospective study and a literature overview. G. Ital. Dermatol. E Venereol..

[40] Zhao C.Y., Hwang S.J.E., Wakade D., Carlos G., Anforth R., Fernández-Peñas P. (2017). Melanocytic lesion evolution patterns with targeted therapies and immunotherapies for advanced metastatic melanoma: An observational study. Australas. J. Dermatol..

[41] Tripepi G., Jager K.J., Dekker F.W., Zoccali C. (2010). Selection bias and information bias in clinical research. Nephron Clin. Pract..

