# Supplementary material for: In-Depth Characterisation of Real-World Advanced Melanoma Patients Receiving Immunotherapies and/or Targeted Therapies: A Case Series
Source: Cancers (Basel). 2022 Jun 4;14(11):2801. doi: 10.3390/cancers14112801 (PMC9179437; doi:10.3390/cancers14112801)
Supplement: Supplementary file 1 [file cancers-14-02801-s001.zip › cancers-1753556-supplementary.pdf]

Supplementary Table S1. Baseline sociodemographic and clinical characteristics (Frequency, column percentages) of participants with and without longitudinal follow up (Frequency, row percentages).

	N= 41 (%)	No follow up N=13 (32%)	Longitudinal Follow up N= 28 (68%)
**Socio-demographic characteristics**			
**Age[1]:**			
**Category**			
≤65 years	21 (51)	8 (38)	13 (62)
>65 years	20 (49)	5 (25)	15 (75)
**Gender**			
Male	26 (63)	9 (35)	17 (65)
Female	15 (37)	4 (27)	11 (73)
**Education**			
High school or less	18 (44)	6 (33)	12 (67)
Post high school qualification	23 (56)	7 (30)	16 (70)
**Environmental exposure**			
**Occupations since leaving school**			
Mainly Indoors	18 (44)	5 (28)	13 (72)
Mainly Outdoors/ both indoor and outdoor	23 (56)	8 (35)	15 (65)
**Overall sports and leisure activity**			
Mainly Indoors/ both indoor and outdoor	13 (32)	3 (23)	10 (77)
Mainly Outdoor	28 (68)	10 (36)	18 (64)
**Phenotype characteristic**			
**Naevi Count (>5mm)**			
Median (range)	-	-	7 (1-59)

**Natural hair colour at age 21**			
Red/Auburn/Blonde/Light Brown	21 (51)	5 (24)	16 (76)
Dark Brown/Black	20 (49)	8 (40)	12 (60)
**Innate skin colour**			
Fair	31 (76)	9 (29)	22 (71)
Medium or olive	10 (24)	4 (40)	6 (60)
**Facultative skin colour**			
Fair	16 (39)	3 (19)	13 (81)
Medium or olive	25 (61)	10 (40)	15 (60)
**Freckling score**			
Nil/Mild (0–6)	18 (44)	5 (28)	13 (72)
Medium/Severe (7-12)	23 (56)	8 (35)	15 (65)
**Body mass index (kg/m^2)**			
Normal (≤24.9)[2]	16 (39)	5 (31)	11 (69)
Overweight (25.0-29.9)	10 (24)	4 (40)	6 (60)
Obese (≥30.0)	15 (37)	4 (27)	11 (73)
**Clinical characteristics**			
**Number of comorbidities**			
None	5 (12)	2 (40)	3 (60)
1	10 (25)	4 (40)	6 (60)
2 or more	26 (63)	7 (27)	19 (73)
**Comorbidities[3]**			
Hypertension			
Yes	14 (34)	4 (29)	10 (71)
No	27 (66)	9 (33)	18 (67)
Hypercholesterolemia or hyperlipidemia			
Yes	10 (22)	2 (20)	8 (80)
No	31 (78)	11 (35)	20 (65)

Cardiovascular disease			
Yes	8 (20)	2 (25)	6 (75)
No	33 (80)	11 (33)	22 (67)
Diabetes mellitus			
Yes	8 (20)	2 (25)	6 (75)
No	33 (80)	11 (33)	22 (67)
**Melanoma history**			
**Number of primary melanomas (n= 39)**			
1	22 (54)	8 (36)	14 (64)
2-7	17 (41)	4 (24)	13 (76)
**Time since diagnosis of most recent primary melanoma (n= 36)**[4]			
≤5 years	21 (58)	8 (38)	13 (62)
5 to ≤10 years	11 (30)	2 (18)	9 (82)
≥11 years	4 (12)	1 (25)	3 (75)
**Time since diagnosis metastatic melanoma**			
≤1 year	16 (39)	10 (62)	6 (37)
2 to 3 years	13 (32)	0	13 (100)
≥4 years	12 (29)	3 (25)	9 (75)
**Melanoma Stage**			
Stage III	5 (12)	0 (0)	5 (100)
Stage IV	36 (88)	13 (36)	23 (64)
**Brain Metastasis**			
Yes	9 (22)	4 (44)	5 (56)
No	32 (78)		23 (72)
		9 (28)	
